# Microwave-Assisted Synthesis of Nickel Oxide Nanoparticles Using *Coriandrum sativum* Leaf Extract and Their Structural-Magnetic Catalytic Properties

**DOI:** 10.3390/ma10050460

**Published:** 2017-04-26

**Authors:** Ramakrishnan Azhagu Raj, Mohamad S. AlSalhi, Sandhanasamy Devanesan

**Affiliations:** 1 Department of Animal Science, Manonmaniam Sundaranar University, Tirunelveli 627 011, Tamil Nadu, India; drazhaguraj@gmail.com; 2 Research Chair in Laser Diagnosis of Cancers, Department of Physics and Astronomy, College of Science, King Saud University, Riyadh 11451, Saudi Arabia; sdnesan1981@gmai.com; 3 Department of Physics and Astronomy, College of Science, King Saud University, Riyadh 11451, Saudi Arabia

**Keywords:** microwave method, X-ray diffraction, electron microscopy, styrene and benzaldehyde, antimicrobial activity

## Abstract

In this paper, using *Coriandrum sativum* L., a leaf-extracted, assisted microwave method (MM) was used to synthesize nickel oxide formation. We synthesized nickel oxide nanoparticles (NiO) with a crystal size in the range of 15–16 nm by a *Coriandrum sativum* leaf-assisted microwave method (LAMM). The synthesized materials show that an X-ray diffraction (XRD) study confirmed the formation of a single phase structure exhibiting a crystallite size in the range of 15–16 nm using Scherrer’s method. The nickel oxide prepared by the MM had a surface area of 60.35 m^2^/g, pore volume of 0.9427 cm^3^/g and an average pore diameter of 13.27 Å. Surface morphology was analyzed by the scanning electron microscope (SEM), X-ray photoelectron spectroscope, Brunauer-Emmett-Teller (BET) analysis, and the vibrating sample magnetometer (VSM). Catalytic activity (CA) tended toward the oxidation of styrene to benzaldehyde. The inexpensive catalyst tested is likely effective as a catalyst due to synergistic interactions between metal oxides with high dispersion. In comparison with other findings, LAMM is easy and eco-friendly. The current study obtained nanocrystalline NiO that was suitable for potential applications in catalysis. The synthesized NiO could potentially be used in therapeutic field due to their competent antibacterial activity.

## 1. Introduction

Currently, semiconductor nanostructures metal oxide materials have established considerable influence owing to their novel properties that differ from their individual components [1]. In the context of material science applications, metal oxides are processed to be altered in material morphology, and the study of these oxides plays a vital role in the capability for manipulating the basic properties of these materials [2]. Transition metal oxides (TMOs) are based on such properties, and this enables these materials to deliver an innumerable number of applications, such as radiation detectors in the ranges required for medical diagnostic imaging, nuclear medicine, and industrial inspection [3]. Nickel oxides (NiO) biological fields are those that include magnetic resonance imaging (MRI) enhancement, magnetic storage data, ferrofluids for the removal of impurities in biological samples, catalysis, drug delivery, sensors, and pigments [4,5]. Metal oxide sizes and shape-dependent properties within personalized morphologies and patterns have allowed scientists to prepare metal oxide nanostructures with controlled sizes and morphological structures. It is necessary for the preparation of metal oxides to occur using simple, effective and inexpensive methods [6,7].

Several soft chemistry processes, such as co-precipitation, sol-gel, hydrothermal, molten salt synthesis, and pyrolysis methods, for covered precursors of double hydroxide and many coordination compounds, have been developed for the preparation of nickel oxides [8,9,10,11,12,13]. Currently, the plant-extracted microwave method (MM) route has attracted significant interest in fabricating homogeneous, nano-sized, functional materials because of its cheap and inexpensive precursors and short preparation time [14,15]. This organic precursor provides a basis for redox reactions to occur during combustion methods. Amongst the numerous controller limitations in a combustion method, fuel plays a large role in determining the morphology and phase formation of the final product. The end product surface area, distribution size and particle accumulation mainly depends on the combustion temperature, which is related to natural fuel and fuel-to-oxidant-dependent ratios. Most recently, plant extracts have also been used as both a reducing and a gelling agent for the synthesis of mixed metal oxides. The plant extract plays a role as a fuel, and also has an organizing action, apprehending the metal ions present in the amylose helix of the plant extract, and obstructing the separation of metal oxides synthesized through the green method, which has been reported previously [14,15,16,17,18,19].

The parsley plant *Coriandrum sativum* is used in garlands for social and religious functions. In South India, it is mainly used in food preparations. It is native to India and Mexico, and also distributed in South East Asia and Europe. Garden parsley is a biennial plant in temperate climates, and also an annual in the subtropical and tropical areas. In the present study, parsley is a source of flavonoids, antioxidants, folic acid, vitamins K, C, and A, and apigenin. Excessive ingestion of parsley by pregnant women should be avoided. While it is safe in normal quantities, excessive amounts consumed may have adverse uterotonic effects [20].

The plant extract plays not only a role as a fuel, but also has a coordinating action, capturing the involved metal ions in the amylose helix of the extract in well-defined sites, and impeding the separation of metal oxides. An attractive subject of this research field is the non-polluting and controlled synthesis of oxide materials, which occurs at a low cost, and using natural compounds as raw materials, and also as active ingredients in the nanosized metal oxides. Most recently, Klinkaewnarongi et al. reported for the first time the synthesis of metal oxide nanoparticles by a simple method using a different plant extract solution. To the best of our knowledge, this biosynthesis route has not been extended to the preparation of Ag, Au, and transition metal oxides [21,22].

Experimental conditions play a very important role in determining the shape, size, purity, and hence, the modification of nanomaterial properties. In contrast with the transformation of nanomaterials into larger structures by simple etching processes, it helpful for control the reaction conditions by removing transformed material from a mixture, as well as reacting the samples to modify larger materials into smaller and simpler structures. Etching processes not only provide a potential route for secondary applications of nanomaterials, but may also serve to narrow size dispersion. Carbonyl compounds can be separated into two kinds: aldehydes and ketones. These are significant raw materials and intermediates for the production of different chemicals, such as dyes, medicines, and perfumes. One of the main methods for aldehyde and ketone production is the selective oxidation of their corresponding alcohols, with a suitable catalyst that plays an important role in these reactions [23,24].

However, most of these conditions demand a strictly controlled synthesis environment, expensive reagents, and complicated procedures. Therefore, simple and cost-effective conditions via MM for the synthesis of nickel oxides by the utilization of cheap, non-toxic, and environmentally benign precursors are key issues for production. Consequently, nickel oxides prepared by a simple MM, and exhibiting good structural, morphological and magnetic properties are interesting materials for electronic and catalytic applications.

## 2. Results and Discussion

**Figure 1:**
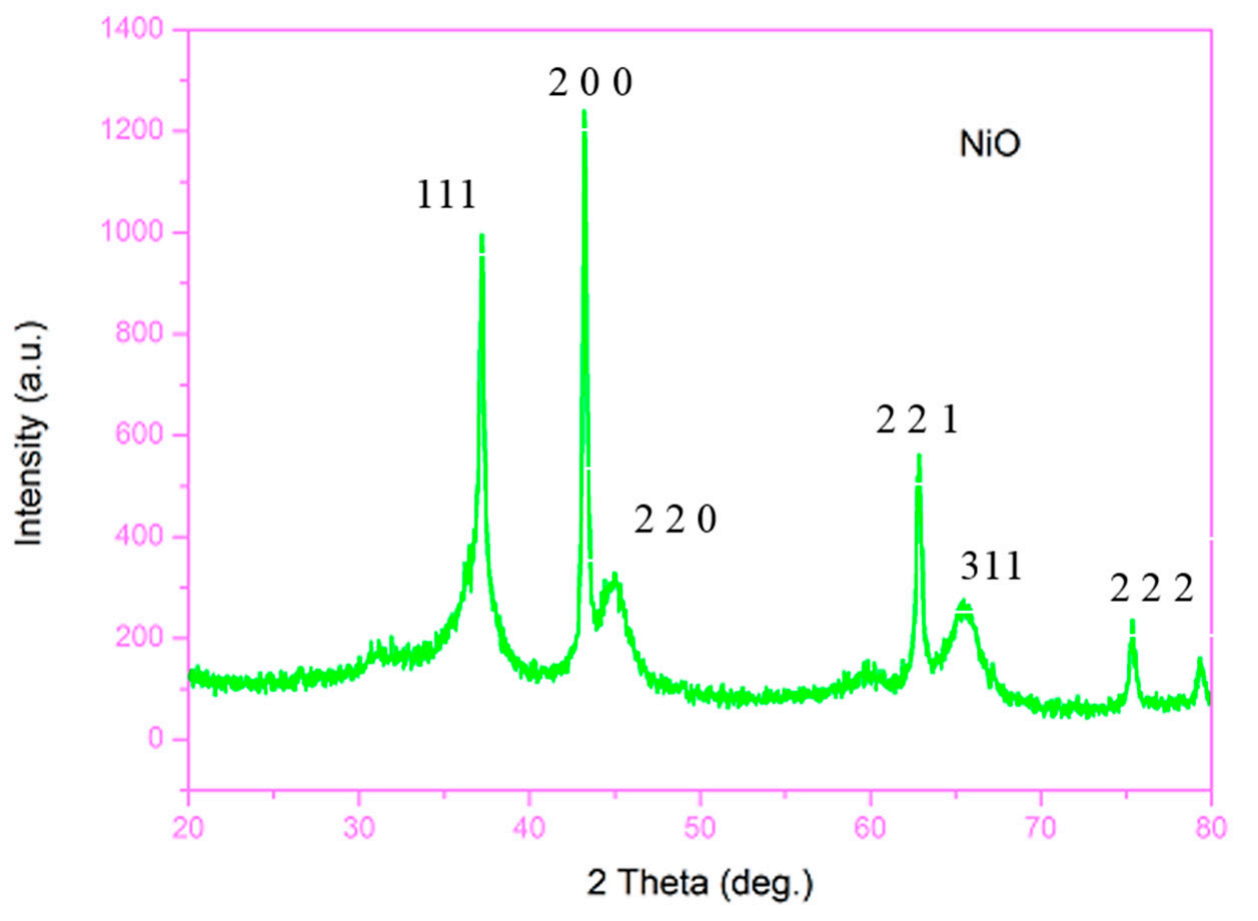
X-ray diffraction (XRD) pattern of nickel oxide.

The structural portion of NiO was obtained by X-ray diffraction (XRD) patterns. Figure 1 shows the XRD outlines of the products obtained as a function of synthesis time within the MM. It can be observed that the NiO shows the presence of characteristic peaks of Joint Committee on Powder Diffraction Standards (JCPDS) No. 78-0643 as well. The average crystallite size was obtained from XRD peaks using the Scherrer equation [25].

$$L=\frac{0.89\lambda }{\beta \cos\theta }$$

where, *L* is average crystallite size (Å), *λ*, the X-ray wavelength (0.154 nm), *β* is full width at half maximum in radians, and *θ* is Bragg’s diffraction angle. The intensity of the XRD peaks on the sharpness denoted high crystallinity and also a large crystallite size. The average crystallite size of the catalyst was 15.66 nm (Figure 1).

The existence of strong and sharp diffraction peaks at 2*θ* values located at 37.451, 43.801, 45.532, 62.171, 69.221, and 75.761, corresponding to (1 1 1), (2 0 0), (2 2 0), (2 2 1) (3 1 1) and (2 2 2) planes, indicated the formation of a pure cubic nickel oxide. For the NiO synthesized by microwave method, small peaks of Ni phase on 37.451, 43.801 and 62.171, corresponding to (1 1 1) (2 0 0) and (2 2 1) crystal planes with fcc metallic nickel, were observed [26].

**Figure 2:**
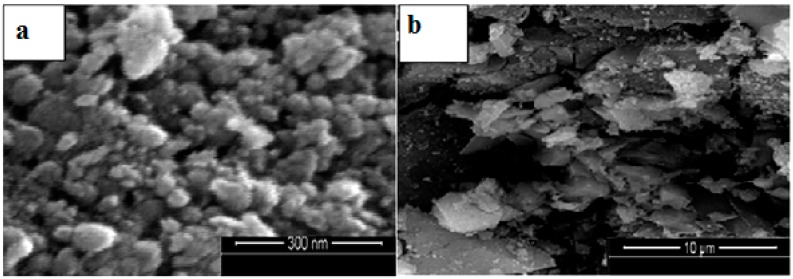
(**a**,**b**) High Resolution Scanning Electron Microscope (HR-SEM) images of nickel oxide.

The surface morphology microscopy (SMM) imaging was performed on the NiO produced via MM. The images showed that the product consisted of nanoparticles in high quantity, homogeneousness and with uniformly distributed grains (Figure 2a,b). The development of modern technology through the MM has recently received increasing attention from the scientific community for the preparation of metal oxides, which can be attributed to the release of large amounts of gas during the combustion process. The observed nanoparticles aggregation of nano- and microstructures was mainly due to the presence of magnetic interactions among the materials. In the microwave route of production, the *Coriandrum sativum* leaf extract and nickel nitrate solution were homogeneously mixed at the atomic scale, due to the collision and friction of uniformly distributed microwaves. It is well known that grain size and morphology of samples are dependent on the temperature and gases generated, due to decomposition during the combustion reaction. Microwave ovens produce enormous amounts of heat that are distributed homogeneously through the interior of materials, and this caused rapid combustion with a higher amount of gas (N_2_, NO_2_, CO_2_ and H_2_O) release, and for the formation of nano-sized particles. In this method, the increase in the particle size was due to the growth of fine nanoparticles by solid state diffusion, thereby reducing the surface area of the nanoparticles to obtain better crystalline structure and homogeneity.

From the scanning electron microscope (SEM) images, the development of the nano-sized spherical and uniform particles with an extraordinary tendency of combination and aggregation was observed. These aggregates were almost similar throughout, thus indicating uniform particle dimension (shape and size). The nanoparticle and crystallite size observed by high resolution (HR)-SEM and XRD respectively may have been different as the crystallite size obtained from XRD was the size of coherently diffracting domains within the nanoparticles [27].

**Figure 3:**
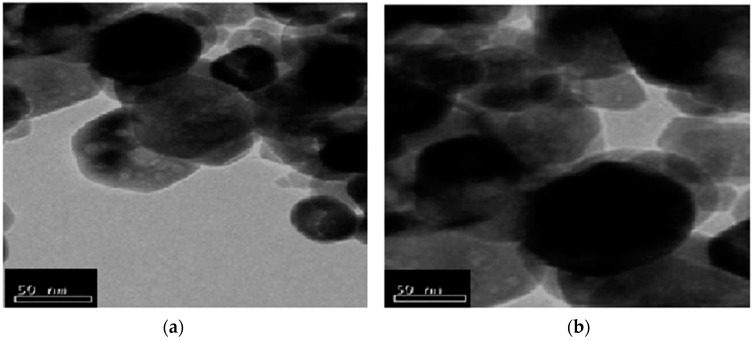
(**a**,**b**) High Resolution Transmission Electron Microscopy (HR-TEM) images of NiO prepared by the microwave method.

To afford additional suggestions in the formation mechanism, transmission electron microscopy (TEM) analysis was conducted for NiO-MM. Direct evaluation of the TEM images showed that the uncapped NiO-MM nanoparticles were aggregated, whereas the plant extract-capped nanoparticles were well separated. The particles were found to be almost spherical (Figure 3a,b), with a homogeneous distribution. The aim of the current study to solubilize and stabilize NiO-MM was achieved. Calculation of size distribution by image J software (https://imagej.nih.gov/ij) based on the measurement of 100 NiO crystallites from TEM images yielded a nearly normal distribution curve with a maximum size of around 14–17 nm. TEM imaging showed NiO-MM that the nanoparticles [NPs] were highly crystalline, with a d-spacing of 0.231 nm. The d-spacing was in perfect correlation with JCPDS No. 78-0643 from XRD measurements. The presence of NiO was due to the TEM gold grid. No impurities were detected in the NiO samples. SEM and TEM images were cleared out due to gold coated on the samples before commencing TEM imaging.

**Figure 4:**
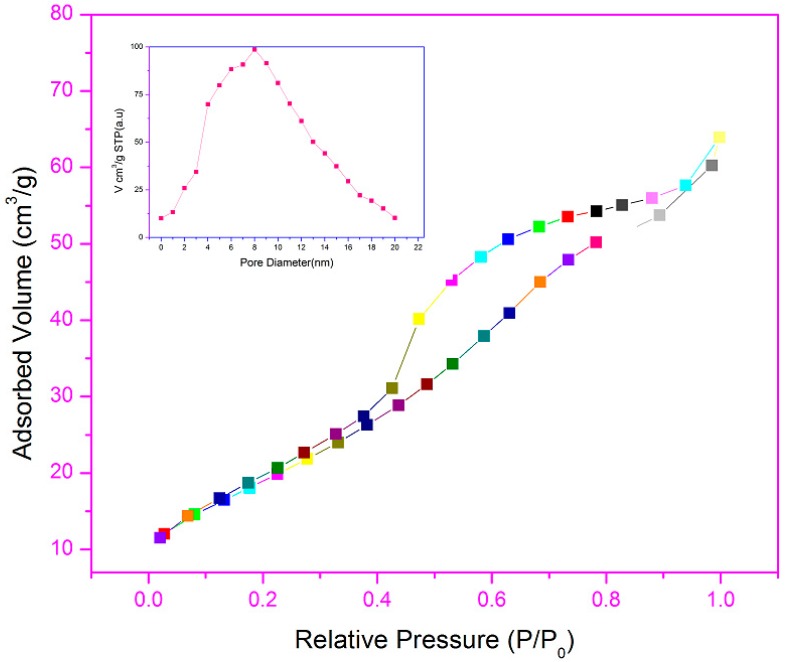
Nitrogen adsorption/desorption isotherm of nickel oxide.

Full nitrogen absorption/desorption isotherm distribution curves of NiO are shown in Figure 4. Nickel oxide-specific surface areas (S_BET_) composed within pore radius (Rp) and pore volume (Vp) were calculated. The nickel oxide prepared by the MM had a surface area of 60.35 m^2^/g, a pore volume of 0.9427 cm^3^/g, and an average pore diameter of 13.27 Å. Hence, it was assumed that the high surface area of nickel oxide could allow for catalytic activity. From the Barrett-Joyner-Halenda (BJH) pore size distributions (inset in Figure 4), we found that all the studied samples showed a single peak in BJH pore size distributions.

**Figure 5:**
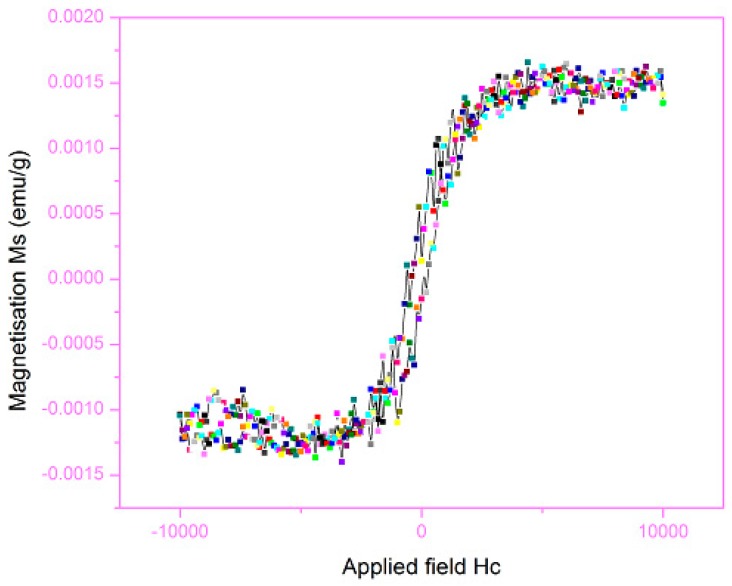
Room-temperature magnetic hysteresis loops of nickel oxide.

Figure 5 shows that nickel oxide produced material with high organizational structure at a low temperature (5 K). Magnetization using an external applied magnetic field ranging between 0 and ±5000 Oe was applied. As clearly shown, the difference of magnetization as a function of the applied field produced a contracted cycle, and the experimental hysteresis loops gave a representative performance of soft magnetic materials. The saturation magnetization (Ms), remanence magnetization (Mr), and the coercivity (Hc) values of these ferrites were 65.23 (Ms) emu/g, 9.21 (Mr) emu/g and 60.34 (Hc) Oe, respectively. It is well known that the magnetic properties of any nanomaterial are strongly dependent on the preparation method, the shapes and sizes of their particles, and their crystallinity [28,29].

**Figure 6:**
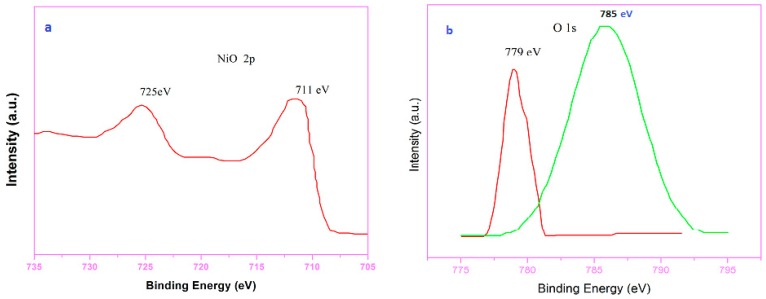
X-ray Photoelectron Spectroscopy (XPS) spectra of (**a**) Ni 2p; and (**b**) O 1s.

The oxidation state of Ni, its arrangement, and the purity of the synthesized NiO were additionally investigated with the help of X-ray photoelectron spectroscopy (XPS) analysis. The resolution spectrum of Ni 2p is given in Figure 6. The nickel 2p orbital spectra at Ni 2p 3/2 and 2p 1/2 produced binding energy values of 725.8 eV and 711.4 eV, respectively; 779 eV and 785 eV were obtained from the spectra oxygen 1s orbital. These XPS results indicated the formation of nickel oxide.

The reaction progress was investigated under the influence of a catalyst; NiO, 0.3 g; styrene, 5 mmol; acetonitrile, 5 mmol; H_2_O_2_, 5 mmol; a temperature of 60 °C, and a time of 6 hr. Owing to the high Lewis basic nature of the nickel oxide, the catalyst contains an equal distribution of Lewis basic sites and the Brønsted acidic sites. The oxidant, H_2_O_2_, has the capability to adsorb on the surface of the catalyst by the Brønsted acidic nature of the catalyst, and the oxygen from the oxidant release to the substrate, due to its Lewis acidic nature. Thus, this catalytic reaction involves conversion of styrene to benzaldehyde [30].

The reactive sites would favor the formation of benzaldehyde as a major product in agreement with the experimental results, and conversion of styrene to benzaldehyde was 83% efficient with 92% selectivity. This demonstrated that the benzaldehyde was formed through direct oxidation cleavage of the C=C bond of styrene, and not through fast conversion of styrene oxide to benzaldehyde. The advantage of the inexpensive catalyst was likely due to synergistic interaction between the metal oxides with high dispersion. This inexpensive procedure might be of interest for the selective oxidation of styrene. Additional research will highlight additional catalytic applications.

**Scheme 1:**
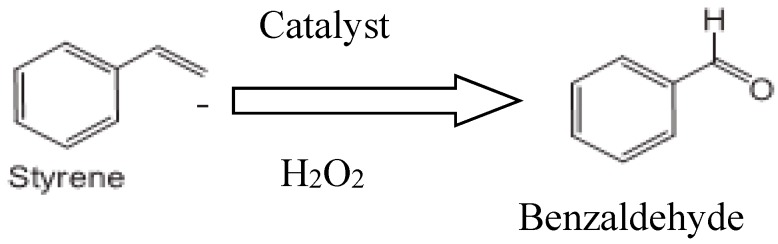
Catalytic reaction of styrene to benzaldehyde.

**Scheme 2:**
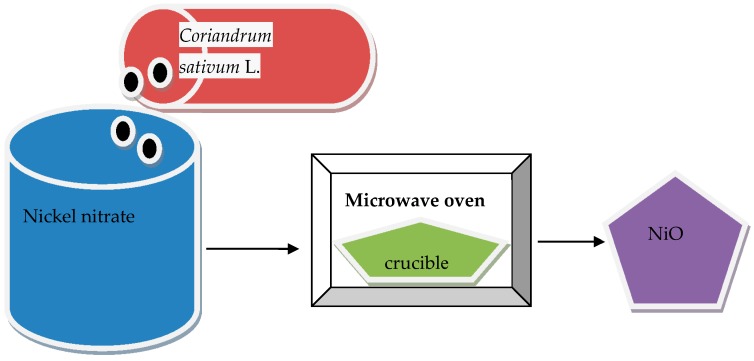
Microwave synthesis for nickel oxide.

The role played by various reaction parameters in determining catalytic efficiency is well recognized, and each parameter has an optimum value for attainment of maximum activity. The NiO catalyst system has been used frequently in liquid phase oxidations, and though catalyst structural stability, morphology, crystalline structure, and surface composition has been obtained after hundreds of hours of testing, knowledge from these types of studies are very limited in for liquid phase reactions. It is well known that NiO catalysts are structurally sensitive to catalytic activation and reaction conditions. A portion of the active sites connecting the catalytic reaction mechanism has been shown in Scheme 1. Till today, no appropriate mechanism for this reaction has been developed in converting styrene to benzaldehyde. The reasonable mechanism (Scheme 2) for the reaction includes NiO interactions, in analogy to other recommended mechanisms for metal oxide-catalyzed reactions [31].

In previous work, sol-gel prepared NiO nanosheets possessed better exposed surfaces suitable for active sites, for the adsorption of reactants in catalytic reaction used for the conversion of benzyl alcohol, in which 70% of benzaldehyde was converted, with 100% selectivity. Therefore, it is observable that the activity of NiO catalysts depends on the preparation method. The MM along with NiO has shown very good conversion in the present study became of the above stated reasons. It may be noted that not only the conversion of benzyl alcohol, but also of benzaldehyde had higher selectivity for samples prepared by the MM [32].

Antibacterial properties of nickel oxide nanoparticles were tested successfully on human Gram-positive and Gram-negative bacterial pathogens. Zones of inhibition were formed, indicating that the nickel oxide nanoparticles synthesized in this process had average activity against the Gram-positive and Gram-negative bacteria (Table 1). The antibacterial activity of the nickel oxide nanoparticles altered Gram-positive and Gram-negative bacterial cell membrane morphology, potentially through interacting with phosphorous- and sulfur-containing compounds such as DNA, and inactivating bacterial enzymes [33]. The nickel nanoparticles may have acted to repress the replication of DNA and inactivate cellular proteins after treatment [34].

Another reason for the antibacterial properties of the nanoparticles would be the release of Ni ions from the NiO nanoparticles, which would have had an additional contribution to the bactericidal efficiency of nickel oxide nanoparticles [35,36,37]. In this study, we synthesized NiO crystallites with a size range of 15–16 nm, using the *Coriandrum sativum* LAMM. In comparison with other findings, this method is easy and eco-friendly. Several researchers have also used the plant or its parts as reduction and stabilization agents for non-material synthesis. Date palm syrup can be used as a reducing agent for the synthesis of nickel oxide nanoparticles with a diameter of 15–16 nm [36]. NiO nanoparticles with a particle size of 15 nm were synthesized by a simple combustion method [37].

In the MM, microwave energy was used to trigger nucleation growth of nickel nitrate and plant extract, Ni cations within a very short time, to form nanocrystalline fine particles, which were homogeneously dispersed. The results confirmed that the microwave method approach is fast and easy, and results in well-formed crystalline nanomaterials within a few minutes, without the need for calcination. NiO nanostructures have been synthesized by a microwave approach in the presence of olive oil, with a NiO nanoparticle size range of 10–20 nm [38]. Nickel oxide nanoparticles with a size of 25 nm have also been synthesized via a decomposition method [39].

In situ synthesis of metal oxide NPs using plant extract is more simple, fast, and economical than combustion methods, and it produces affordable nano-sized metal oxides with tunable sizes or morphologies, as shown from optical, magnetic, and surface analysis. Recently, agglomerated nanoparticles of metal oxides have been synthesized using methods at low-temperature [40]. In this study, the obtained nanocrystalline NiO material were suitable for potential applications in catalysis.

### Comparison of NiO Activity with Other Reported Methods

The tested method promises to be one of the most versatile of preparation methods, as it enables the control of particle properties, such as particle size, morphology, homogeneity, and surface area. These unusual properties have been attributed to the extremely small sizes and the high specific surface areas of the nanoparticles. Plant extract is non-polluting, and thus enables controlled synthesis of nanomaterials at low cost, since natural compounds are used as raw materials and active ingredients. Combustion synthesis is now considered to be one of the most accessible, rapid, and economic soft methods for the synthesis of simple and mixed nanometal oxides, as confirmed in the present study through XRD, SEM, and analysis of optical properties). Table 2 shows the comparison of structure and particle size of NiO prepared in the present work, and those synthesized by other chemical methods [41,42,43].

## 3. Materials and Methods

### 3.1. Materials

Analytical grade reagents, such as Ni(NO_3_)_2_·6H_2_O, were used as raw materials (Merck Chemicals, Mumbai, India). They were used as received without any further purification. Leaves of *Coriandrum sativum* were collected from local agricultural fields at Kanchipuram, Tamil Nadu, India.

### 3.2. Preparation of Plant Extract

In the first process, approximately 100 g of leaves were thoroughly washed to properly remove dust. Using a very sharp knife, the tip of the leaf was cut carefully to produce roughly pointed edges on both sides of the leaf. It was ensured that both sides of the leaf were cut all the way from top to bottom. In the second process, the leaf portion (lamina) was separated from the leaves, which were finely cut and dissolved in 500 mL of deionized water, and stirred at 50–60 °C for 45 min to obtain a clear solution. The resulting extract was used as the plant extract solution of *Coriandrum sativum* L.

### 3.3. Synthesis of Nickel Oxide by Microwave Methods

Microwave methods depend on the principle of propellant chemistry in the construction of redox mixtures, in which the nickel nitrate [Ni(NO_3_)_2_·6H_2_O] and fuel from *Coriandrum sativum* L. extract solution was used as a reducing reactant. Stoichiometric amounts of nickel nitrate (0.581 g) were dissolved in the water under constant stirring using a magnetic stirrer. *Coriandrum sativum* L., extract solution was dissolved separately in 30 mL of deionized water, and poured into a silica crucible and stirred for 15 min to obtain a clear solution. This was placed in a domestic microwave oven (3.45 GHz, 950 W) for 10 min. Initially, the solution reached boiling point and underwent evaporation, followed by decomposition with the evolution of gases. When the point of spontaneous combustion was reached, it vaporized the solution instantly and became solid. The MM has attracted attention in producing homogeneous, agglomerated, multi-component metal oxide ceramic powders, because of its inexpensive precursors and short preparation time. The microwave procedure produced nickel oxide powders in a microwave oven operated at a power of 950 W, and at temperatures ranging from 150 to 400 °C, resulting in the formation of NiO within 10 min. The solid product obtained was centrifuged twice at 3000 rpm for 20 min, thoroughly washed with alcohol, and dried at 120 °C for three hours. The resultant dried precursor was crushed into powder and stored in an air tight container for additional analysis [44].

This procedure was repeated for nickel oxide preparation. We conclude with a simple scheme which describes the plant extract processes on prepared by transition metals oxide (Scheme 2). The representative materials in the manuscript can be changed for different fuels, while following the same method, by changing the concentration of constituents. This method allows nano-sized materials in bulk quantities to be synthesized in comparatively less time, and in various structures. There are many applications to the novel method proposed in this study.

### 3.4. Characterization

Structural characterization of nickel oxide was performed by using a Philips X’pert X-ray diffractometer (Bruker AXS GmbH, Essen, Germany) with Cu Kα radiation at λ = 1.540 Å. Morphological studies and energy dispersive X-ray analysis of nickel oxide was performed with a Jeol JSM6360 HR-SEM (JEOL, Tokyo, Japan). Morphology and average particle size were determined by TEM. XPS analysis was performed on an ESCA-Lab250 electron spectrometer (Thermo Scientific Corporation, Waltham, MA, USA) using monochromatic 150 W Al Kα radiations; the pass energy for the narrow scan was 30 eV. Nitrogen adsorption–desorption isotherms of the samples were measured by using an automatic adsorption instrument (Quantachrome Quadrawin gas sorption analyzer, Quantachrome, FL, USA) for the purpose of obtaining surface area and total pore volume. Magnetic properties were examined by a vibrating sample magnetometer (VSM) (Lake Shore Cryotronics, Inc., Westerville, OH, USA) at room temperature in an applied magnetic field sweeping from −10,000 to +10,000 Oe using a PMC Micro Mag 3900 model VSM equipped with a 1 T magnet. The styrene-oxidized product obtained after the catalytic reaction was collected and studied using the Agilent gas chromatography (GC) technique. The column used for the study was a polyethylene glycol -dibromo[DB] wax column- capillary column) of length 30 mm and with helium used as the carrier gas.

### 3.5. Antibacterial Activity

Antibacterial activity was assayed using the Agar well diffusion method (AWDM). Petri plates (9 cm) were prepared with 20 mL sterile Muller-Hinton agar (Hi-Media, Mumbai, India). Wells were made using a sterile cork-borer under aseptic conditions. Four clinical strains used for the present study, *Staphylococcus aureus*, *Proteus vulgaris*, *E. coli*, and *Pseudomonas aeruginosa*, these organisms were collected from Villupuram Medical College, Villupuram Tamilnadu, India. Nickel oxide nanoparticles with various concentrations (10, 20 and 40 µg/mL) were added to the respective wells. Chloramphenicol (0.1%; Hi-Media, India) and double distilled water were used as a positive and negative control, respectively. After incubation for 24 hr at 37 °C, a clear zone of inhibition formed in each well, evidence of antibacterial activity. The diameter of the inhibition zone was measured in millimeters using a Hi-Media ruler. Each test was performed in triplicate [45].

## 4. Conclusions

*Coriandrum sativum* leaf plant-extracted assisted MM was used to synthesize nickel oxide, and it was used selectively in the oxidation of styrene. A nickel oxide-crystallized single-phase was obvious from XRD patterns, with a lattice constant and a cell volume matching the standard spinal pattern. It is expected that this work may be utilized for a clean catalytic procedure in the perfumery industry, and the evaluation of benzaldehyde, where other products and benzoic acid can be avoided. In this study, the obtained nanocrystalline NiO material was suitable for potential applications in catalysis. Nickel oxide nanoparticles would be useful in the medical field, due to their competent antibacterial activity.

## Figures and Tables

**Table 1:** Antibacterial activity of nickel oxide nanoparticles.

Pathogens	Zone of Inhibition Diameter (mm)				
	10 µg/mL	20 µg/mL	40 µg/mL	Positive Control Chloramphenicol (0.1 %)	Negative Control
*Staphylococcus aureus*	10 ± 0.3	11 ± 0.9	13 ± 0.1	24 ± 0.9	0
*Proteus vulgaris*	09 ± 0.1	10 ± 0.6	12 ± 0.4	19 ± 0.9	0
*E. coli*	12 ± 0.3	13 ± 0.4	15 ± 0.6	25 ± 0.3	0
*Pseudomonas aeruginosa*	08 ± 0.1	11 ± 0.3	13 ± 0.7	23 ± 0.6	0

**Table 2:** Comparison of NiO activity with other reported methods.

Synthesis Method	Particle Size (nm)	Morphology	Reference
Present study	15–17	nanoparticles	-
Polymerization method	26–35	nanoparticles	[41]
Sonochemical method	24–28	nanoparticles	[42]
Microwave method	20–25	nanoparticles	[43]
